# Cytoplasmic α-Amylase AmyA Modulates Maltodextrin/Glycogen Metabolism in *Escherichia coli*

**DOI:** 10.4014/jmb.2606.06044

**Published:** 2026-09-04

**Authors:** Ji -Yun Kwak, Yuna Kang, Jong-Tae Park

**Affiliations:** 1 Department of Food Science and Technology, Chungnam National University, Daejeon 34134, Republic of Korea; 2 Department of Crop Science, Chungnam National University, Daejeon 34134, Republic of Korea; 3 CARBOEXPERT Inc., Daejeon 34134, Republic of Korea; 4 National Agrobiodiversity Center, National Institute of Agricultural Sciences, Jeonju-si 54874, Republic of Korea

**Keywords:** Cytoplasmic α-amylase, AmyA, *Escherichia coli*, Maltose/Maltodextrin metabolism, Glycogen metabolism

## Abstract

Cytoplasmic α-amylases represent an unusual subgroup of GH13 enzymes found across diverse bacterial genera, yet their physiological roles remain elusive. Among them, AmyA from *Escherichia coli* is the most well-known intracellular α-amylase, but its roles in maltose/maltodextrin utilization and glycogen metabolism have not been clearly characterized. AlphaFold-based domain structure and phylogenetic analyses revealed that AmyA retains the canonical A/B/C core domain fold similar to extracellular bacterial α-amylases but lacks a signal peptide, pro-region, and additional C-terminal domains. Based on its structure, AmyA represents a minimal intracellular α-amylase lineage distinct from classical extracellular enzymes. AmyA hydrolyzed linear maltodextrins, producing short maltooligosaccharides including maltose and maltotriose, but showed no detectable activity toward cyclodextrins. Deletion of *amyA* did not affect the growth rate in glucose minimal medium but caused a pronounced growth delay when maltose was used as the sole carbon source. Glycogen accumulation was higher in the Δ*amyA* mutant under both carbon-source conditions. In maltose minimal medium, several maltose-utilization genes showed lower transcript abundance in the Δ*amyA* mutant, accompanied by changes in the expression of glycogen-related genes. Together, these findings suggest that AmyA contributes to intracellular maltodextrin processing and is associated with changes in maltose utilization and glycogen metabolism in *E. coli*.

## Introduction

α-Amylases are widely distributed hydrolytic enzymes that catalyze the degradation of starch and related polysaccharides. Most bacterial α-amylases belong to glycoside hydrolase family 13 (GH13), which share a characteristic three-domain architecture consisting of the catalytic (β/α)_8_-barrel A-domain, an inserted B-domain, and a C-terminal β-sandwich C-domain [1]. These enzymes are generally synthesized with N-terminal signal peptides and exported to the periplasm or extracellular space, where they hydrolyze environmental polysaccharides into assimilable oligosaccharides. Consequently, extracellular GH13 α-amylases have been extensively characterized across diverse microorganisms, and their structure-function relationships are well understood.

In contrast, a distinct group of cytoplasmic α-amylases lacking N-terminal secretion signals has been identified in several bacterial genera. Such enzymes have been reported in *Escherichia coli* and *Salmonella* [2], while an intracellular α-amylase from *Lactobacillus acidophilus* was shown to contribute to glycogen metabolism and stress tolerance [3]. An early study characterized the chromosomally encoded cytoplasmic α-amylase (AmyA) of *E. coli* and *Salmonella*, describing basic enzymatic and regulatory properties [4]. Despite these observations, the physiological significance of AmyA and its relationship to carbohydrate metabolism remain unclear. Furthermore, homologs of AmyA are widely distributed among diverse bacterial taxa, particularly within members of the Enterobacterales, suggesting that cytoplasmic GH13 α-amylases may play broader and previously unrecognized roles in bacterial physiology.

Carbohydrate processing in *E. coli* involves two interconnected pathways: the maltose/maltodextrin utilization system and the glycogen metabolic network. Environmental polysaccharides are hydrolyzed into oligosaccharides that can cross the outer membrane [5]. Maltodextrins first cross the outer membrane through LamB and are subsequently transported across the inner membrane by the MalFGK_2_ ABC transporter [6, 7]. In the periplasm, MalS, a periplasmic α-amylase, hydrolyzes incoming malto-oligosaccharides and oligosaccharides smaller than maltohexaose are transported into the cytoplasm for further processing [5, 8-10]. Once inside the cell, maltose and maltodextrins are metabolized by enzymes of the maltose/maltodextrin regulon [11]. A key component of this system is maltotriose, which serves as the inducer of MalT, the master transcriptional activator controlling expression of the *mal* genes [12]. MalQ, a 4-α-glucanotransferase, converts maltose to maltotriose and is essential for growth on maltose medium [13]. Despite extensive knowledge of the maltose regulon, how AmyA contributes to intracellular carbohydrate metabolism remains poorly understood.

Glycogen metabolism is tightly linked to maltodextrin processing because intracellular glucans function as both metabolic intermediates and storage compounds. Disruption of maltodextrin phosphorylase (MalP) results in excessive glycogen accumulation [14], highlighting the close metabolic connection between these pathways. Carbon derived from maltodextrin metabolism can be redirected into glycogen biosynthesis through enzymes encoded by the *glg* operon, which contains genes involved in both glycogen synthesis and degradation [15]. As a result, enzymes that influence intracellular maltodextrin composition are likely to affect both maltose utilization and glycogen homeostasis.

Although AmyA does not contain a MalT-binding regulatory sequence, its cytoplasmic localization suggests that it may influence the pool of short linear maltodextrins associated with MalT-dependent regulation and intracellular carbohydrate metabolism. Because intracellular carbohydrate-processing enzymes can affect multiple metabolic pathways simultaneously, clarifying the role of AmyA is essential for understanding how *E. coli* coordinates maltose utilization with glycogen metabolism.

In this study, the enzymatic properties and substrate preferences of AmyA were examined, and its physiological contribution was evaluated through comparative analysis of growth phenotypes, glycogen accumulation, and transcriptomic responses in a Δ*amyA* mutant and its parental strain. These analyses provide insight into the physiological role of cytoplasmic α-amylase in intracellular maltodextrin utilization and glycogen metabolism in *E. coli*.

## Materials and Methods

### Phylogenetic Analysis

AmyA homologs and representative GH13 α-amylases from diverse bacterial taxa were retrieved from the NCBI database in FASTA format for phylogenetic analysis. Redundant and partial sequences were excluded prior to alignment. Amino acid sequences were aligned using MUSCLE implemented in MEGA 12 with default parameters. The best-fit amino acid substitution model was selected using the model selection function implemented in MEGA 12, and the Whelan and Goldman (WAG) model with gamma-distributed rate variation and invariant sites (WAG+G+I) was selected according to the Bayesian Information Criterion (BIC). Phylogenetic analysis was performed using the Maximum Likelihood method with the WAG+G+I model. The reliability of the inferred tree topology was evaluated by 1,000 bootstrap replicates. The resulting tree was visualized and annotated in MEGA 12 to examine the phylogenetic relationships among AmyA homologs and related GH13 α-amylases.

### Domain Architecture Analysis

The predicted three-dimensional structure of *E. coli* AmyA was obtained from the AlphaFold Protein Structure Database (UniProt ID: P26612). The highest-confidence model provided by AlphaFold was used for structural analysis, and residue-level confidence was interpreted based on pLDDT scores included in the database entry. To assess domain architecture, the AlphaFold model of AmyA was compared with representative GH13 α-amylase structures, including *Bacillus licheniformis* α-amylase (PDB: 1BLI) [16] and *Bacillus stearothermophilus* maltogenic α-amylase (PDB: 1QHP) [17]. Structural alignment was performed in PyMOL 2.5 using the conserved catalytic (β/α)_8_-barrel core as the alignment reference. The positions of the catalytic Asp–Glu–Asp residues were identified and compared among the three structures to evaluate conservation of the catalytic framework [18, 19]. Domain boundaries were assigned based on structural correspondence and secondary-structure continuity: Domain A (residues 1-105 and 209-405), Domain B (106−208), and Domain C (406−495). Domain-colored structural images were generated using PyMOL.

### Cloning, Protein Expression, and Purification

Genomic DNA of *E. coli* K-12 MG1655 was extracted using a genomic DNA extraction kit (G-spin^TM^ Total DNA Extraction Kit, Intron Biotechnology, Republic of Korea). The putative *amyA* gene (g.i.: 946434) was amplified by PCR using forward (*NdeI*, 5′-GGAGTCGACATATGCGTAATCCCACGCTGTTAC-3′) and reverse (*HindIII*, 5′-GCCGGGGAAGCTTTTAAATCACCTCTTCGATAAC-3′) primers. The amplified PCR fragment was digested with restriction enzymes and cloned into the p6xHis119 vector. The recombinant plasmid, designated p6xH*amyA*, was transformed into *E. coli* BL21 (DE3), and the cells were cultured in 1 L of LB broth at 37°C. The cells were collected after 20 h of cultivation, and the cell pellet was resuspended in 100 ml of lysis buffer (300 mM NaCl, 50 mM Tris buffer, pH 7.5, and 10 mM imidazole). The cells were disrupted by ultrasonication (Q500, Qsonica, USA) in an ice bath and centrifuged (13,000 rpm, 4°C, 15 min). The recombinant protein in the supernatant was purified by nickel-nitrilotriacetic acid (Ni-NTA) affinity chromatography (Ni-NTA agarose, Qiagen, Germany).

### Enzyme Activity Assay

The protein concentration of purified AmyA was determined using the Bradford protein assay with bovine serum albumin (BSA, BoboStar, Australia) as a standard. A diluted sample (40 μL) was mixed with 2 mL of the Bio-Rad Protein Assay Dye Reagent Concentrate (Cat# 500-0006, Bio-Rad, USA), and the absorbance was measured at 595 nm using a spectrophotometer (Optizen POP, Mecasys, Republic of Kores).

α-Amylase activity was measured by dinitrosalicylic acid (DNS) assay with soluble starch as the substrate. The reaction mixture (300 μL) containing AmyA, 0.5% substrate and 1 mM CaCl_2_ in 50 mM MOPS buffer (pH 7.0) was incubated at 45°C for 10 min. After the addition of 900 μL of DNS solution, the mixture was boiled for 5 min and cooled to room temperature. The absorbance of the final reaction mixture was measured at 575 nm using a spectrophotometer, and the amount of reducing sugar was calculated using a maltose standard curve.

### Thin-Layer Chromatography (TLC)

For substrate specificity analysis, various substrates were incubated with AmyA, and the reaction products were analyzed by TLC. Maltose (G2), maltotriose (G3), maltotetraose (G4), maltopentaose (G5), maltohexaose (G6), maltoheptaose (G7), soluble starch (Ss), amylose (AL), amylopectin (AP), glycogen (Glg), pullulan (Pul), α-cyclodextrin (α-CD), β-CD, and γ-CD were individually used as substrates at a final concentration of 0.5%. For each substrate, a control reaction without AmyA was prepared under the same conditions. The reaction mixture contained 0.04% sodium azide and 1 mM CaCl_2_ in 50 mM MOPS buffer (pH 7.0). AmyA (67 U/mL) was diluted 50-fold and incubated with each substrate at 35°C for 16 h. The reaction was terminated by boiling for 10 min, followed by centrifugation (13,000 rpm, room temperature, 10 min). The G2–G7 standard was prepared using 100 μL of each substrate at a final concentration of 0.28%. α-, β-, and γ-CD standards were prepared by mixing 200 μL of each 1% solution with 400 μL of distilled water. Standards for the other substrates were prepared using the same reaction mixtures without AmyA. The developing solvent consisted of isopropanol, ethyl acetate, and water (3:1:1). The dipping solution consisted of methanol containing 0.3% (w/v) N-(1-naphthyl) ethylenediamine and 5% (v/v) sulfuric acid. Samples were adjusted to a concentration of 5 mg/mL, and 1 μL of each sample was spotted onto TLC glass plates (TLC Silica Gel 60 F254, 20 × 20 cm; Supelco, Germany).

### Mutagenesis

To disrupt *amyA* in the chromosomal DNA of *E. coli* MC4100, a kanamycin-resistance (Km^R^) cassette from pKD13 was inserted into the *amyA* gene by PCR. A 1.4-kbp DNA fragment containing the *amyA* gene interrupted by the Km^R^ cassette and FLP recognition target (FRT) sites was transformed by electroporation into *E. coli* MC4100 harboring pKD46, which expresses λ Red recombinase [20]. Disruption of the chromosomal *amyA* gene (Δ*amyA*) in transformed *E. coli* MC4100 cells was verified by PCR using homologous and heterologous primer pairs. The Δ*amyA* mutant (MC4100Δ*amyA*) was selected on LB agar plates containing kanamycin.

### Growth in Glucose- or Maltose-based Minimal Medium

*E. coli* MC4100 and MC4100Δ*amyA* were cultured in M63 minimal salt medium (15 mM (NH_4_)_2_SO_4_, 22 mM KH_2_PO_4_, 40 mM K_2_HPO_4_, 1 mM MgSO_4_, 0.025 mM FeCl_2_) containing either 0.5% glucose or 0.5% maltose to compare their growth profiles. Each strain was cultured in 5 mL of minimal medium at 37°C for 12 h and inoculated at 2% (v/v) into 150 mL of the corresponding medium. Optical density at 600 nm (OD_600_) was measured every 1-2 h until the stationary phase to generate growth curves.

### Glycogen Analysis

The wild-type and Δ*amyA* strains were cultured in 150 mL of M63 minimal medium containing 0.5% maltose at 37°C, and growth curves were generated. Cells from both strains were harvested every 1 h at six time points between the early exponential and stationary phases (5 ml per time point). For glycogen extraction, cells were collected by centrifugation at 13,000 rpm for 15 min at 4°C. The cell pellets were resuspended in 10 mL of 50 mM sodium acetate buffer (pH 4.5) and boiled for 10 min. After cooling, the cells were disrupted using an ultrasonic processor equipped with a microtip at 30% amplitude for 7 min. The cell extracts were centrifuged at 10,000 rpm for 10 min at room temperature, and the supernatants were collected. Twenty milliliters of ice-cold 99.9% ethanol was added to each supernatant, and the samples were incubated at −20°C for more than 2 h to precipitate glycogen. After centrifugation at 6,000 rpm for 20 min at 4°C, the supernatant was removed. The resulting pellet was washed with 10 mL of ethanol and then freeze-dried. The dried samples were dissolved in distilled water and mixed with 1 mL of 5% phenol reagent. Concentrated sulfuric acid (5 mL) was added directly to the mixture. The samples were incubated at room temperature for 20 min, and the absorbance was measured at 470 nm for quantitative analysis. Maltose solutions ranging from 0.01 to 0.1 mg/mL were used as standards.

Glycogen side-chain distribution was analyzed by high-performance anion-exchange chromatography (HPAEC) after debranching with isoamylase. Dried samples were dissolved in distilled water at 2 mg/mL and incubated with isoamylase (200 U/mg; 50 mM sodium acetate buffer, pH 3.5; Megazyme International Ireland Ltd.) for 72 h at 40°C to hydrolyze α-1,6-glycosidic linkages. After the reaction, the samples were boiled for 5 min to inactivate the enzyme and filtered through 0.2-μm membranes. Side-chain distribution was analyzed using a DX-500 system (Dionex, USA) equipped with a pulsed electrochemical detector (ED40, Dionex), a CarboPac PA-1 column (250 × 4 mm, Dionex), and a guard column (50 × 4 mm, Dionex). The column was pre-equilibrated with 150 mM NaOH, and the samples were eluted at a flow rate of 1 ml/min using 600 mM sodium acetate in 150 mM NaOH.

### RNA Sequencing

To compare RNA expression levels between the wild-type and Δ*amyA* strains, both strains were cultured in 5 mL of M63 minimal medium containing 0.5% maltose at 37°C. Each strain was inoculated at 1% (v/v) into 200 mL of M63 minimal medium containing 0.5% maltose, and cells were harvested at the late exponential phase. Two biological replicates were prepared for each strain. Total RNA was manually isolated using the TRIzol reagent method. Collected cells were washed with PBS and resuspended in 1 mL of TRIzol reagent. After homogenization, the samples were incubated for 5 min at room temperature, followed by the addition of 200 μL of chloroform and vortexing. The samples were incubated at room temperature for 3 min and centrifuged at 12,000 × *g* for 15 min at 4°C. The aqueous phase was transferred to a new tube containing 500 μL of isopropanol and incubated for 10 min at room temperature. The samples were then centrifuged at 12,000 × *g* for 10 min at 4°C, and the supernatant was discarded. The RNA pellet was washed with 1 mL of 75% ethanol and centrifuged at 7,500 × *g* for 5 min at 4°C. After complete removal of the ethanol, the RNA pellet was dissolved in RNase-free water. RNA quality was assessed based on rRNA band integrity using an Agilent RNA 6000 Nano Kit (Agilent Technologies, USA). Prior to cDNA library construction, 2 μg of total RNA was used for poly(A) mRNA enrichment with oligo(dT) magnetic beads. The purified mRNA was fragmented into short fragments, followed by synthesis of double-stranded cDNA. The cDNA was subjected to end repair and poly(A) addition and ligated to sequencing adapters using the TruSeq RNA Sample Prep Kit (Illumina, USA). Suitable fragments were purified using a BluePippin 2% agarose gel cassette (Sage Science, USA) and used as templates for PCR amplification. The sizes and quality of the final libraries were evaluated electrophoretically using an Agilent High Sensitivity DNA Kit (Agilent Technologies), and the library fragments ranged from 350 to 450 bp. Subsequently, the libraries were sequenced using an Illumina HiSeq 2500 sequencer to generate 100-bp paired-end reads (Illumina). Sequencing and genome-mapping statistics for each biological replicate are provided in Supplementary Fig. S1A.

### Transcriptome Analysis

Low-quality reads were removed according to the following criteria: reads containing more than 10% ambiguous bases (N), reads containing more than 40% of bases with a quality score below 20, and reads with an average quality score below 20. Filtered reads were mapped to the *E. coli* K-12 reference genome (Ensembl release 31) using TopHat v2.0.13.

Transcript abundance was estimated using Cufflinks v2.1.1 with the Ensembl release 31 gene annotation database. Non-coding regions were excluded using the --mask option. Multi-read correction and fragment bias correction were applied to improve quantification accuracy. Expression levels were reported as fragments per kilobase of transcript per million mapped reads (FPKM). To compare transcriptional changes associated with *amyA* deletion, mean FPKM values from biological replicates were used to calculate log_2_ fold changes for genes involved in glycogen metabolism and maltose utilization. Genome-wide differential-expression results, including *p* values and false discovery rate (FDR)-adjusted *q* values, are provided in Supplementary Table S2 and visualized as a volcano plot in Supplementary Fig. S1b.

## Results

### Phylogenetic Position and Structural Characteristics of AmyA

Phylogenetic analysis showed that *E. coli* AmyA clustered with putative cytoplasmic GH13 α-amylases from members of the Enterobacterales, including *Shigella*, *Salmonella*, *Citrobacter*, *Klebsiella*, and *Raoultella* (Fig. 1A). Similar AmyA homologs were also identified in more distantly related bacterial genera, including *Yersinia*, *Serratia*, *Agrobacterium*, *Cytophaga*, a Flavobacteriaceae bacterium, and *Bifidobacterium*, indicating that AmyA-like cytoplasmic GH13 α-amylases are widely distributed among phylogenetically diverse bacterial taxa.

Structural analysis revealed a simplified domain architecture in AmyA relative to extracellular and maltogenic α-amylases. The AlphaFold model of AmyA exhibits the canonical GH13 A-, B-, and C-domain arrangement in a compact form (Fig. 1B). In contrast, *B. licheniformis* α-amylase (1BLI) and the maltogenic α-amylase 1QHP possess signal peptides and/or accessory domains absent from AmyA, whereas 1QHP additionally contains the D- and E-domains characteristic of maltogenic GH13 enzymes (Fig. 1C, D). Despite these differences in domain organization, the catalytic Asp–Glu–Asp residues were conserved among AmyA, 1BLI, and 1QHP, indicating preservation of the catalytic framework characteristic of GH13 α-amylases. The linear domain schematic further highlights these differences, showing that AmyA consists solely of the catalytic A/B/C domains, whereas extracellular and maltogenic α-amylases exhibit more complex domain architectures (Fig. 1E). Together, these results indicate that AmyA retains the conserved GH13 catalytic framework while exhibiting a simplified domain architecture characteristic of intracellular GH13 α-amylases.

### Enzymatic Properties of AmyA

Purified AmyA hydrolyzed linear α-1,4-linked glucans, including amylose and maltooligosaccharides ranging from G4 to G7 (Fig. 2). Maltose (G2) and maltotriose (G3) showed little hydrolysis, while amylopectin and glycogen were also hydrolyzed by AmyA. In contrast, little or no hydrolysis was observed for pullulan or cyclodextrins. The specific activity of AmyA toward soluble starch was 6.3 U/mg protein.

Time-course TLC analysis showed progressive hydrolysis of soluble starch, accompanied by the formation of shorter maltooligosaccharides (Fig. 3). Short maltooligosaccharides, including maltose and maltotriose, were detected as hydrolysis products. Glycogen was also hydrolyzed by AmyA, but the formation of hydrolysis products was less apparent than that observed with soluble starch. Consistent with the substrate specificity analysis, AmyA hydrolyzed linear maltooligosaccharides and generated shorter products, including maltose and maltotriose, without detectable glucose accumulation.

### Carbon Source-Dependent Physiological Role of AmyA

Deletion of *amyA* had little effect on growth in glucose minimal medium, where the growth profiles of MC4100 and the Δ*amyA* mutant were nearly identical (Fig. 4A). In contrast, the Δ*amyA* mutant exhibited a pronounced delay during the early growth phase in maltose minimal medium, although both strains ultimately reached similar final cell densities (Fig. 4B). These observations indicate that the physiological contribution of AmyA is more closely associated with maltose utilization than with glucose metabolism. Intracellular glycogen accumulation was subsequently compared between the two strains at the early stationary phase (Fig. 4C). In glucose minimal medium, the Δ*amyA* mutant accumulated 0.262 mg glycogen/mg protein, compared with 0.158 mg glycogen/mg protein in the wild type. In maltose minimal medium, glycogen accumulation increased in both strains, reaching 0.189 mg glycogen/mg protein in the wild type and 0.338 mg glycogen/mg protein in the Δ*amyA* mutant. Thus, glycogen levels were consistently higher in the mutant than in the wild type under both carbon source conditions, with the greatest accumulation observed in the Δ*amyA* mutant grown on maltose.

To further examine glycogen metabolism during growth, cell growth and glycogen levels were monitored throughout cultivation in G2 medium (Fig. 5). Both strains accumulated glycogen during growth and subsequently degraded glycogen upon entry into stationary phase. However, glycogen levels were generally higher in the Δ*amyA* mutant during cultivation, consistent with the increased glycogen accumulation observed at the early stationary phase.

Analysis of glycogen chain-length distribution revealed differences between the wild-type and Δ*amyA* strains (Fig. 6). Glycogen from the Δ*amyA* mutant showed a higher proportion of side chains in the DP 10–20 range and a lower proportion of longer side chains than that from the wild type. Similar changes in chain-length distribution were observed in both glucose and maltose minimal media. These results suggest that *amyA* deletion is associated with changes in glycogen side-chain distribution.

### Transcriptomic Alterations Associated with *amyA* Deletion

To further investigate the physiological basis of the Δ*amyA* phenotype, transcript abundance of genes associated with maltose utilization and glycogen metabolism was compared between the wild-type and Δ*amyA* strains grown in maltose minimal medium (Fig. 7 and Supplementary Table S1). Analysis of mean FPKM-derived log_2_ fold changes revealed changes in the expression of genes involved in both pathways. Replicate-specific FPKM values and the corresponding differential-expression statistics are provided in Supplementary Table S1, while genome-wide differential-expression patterns and the complete differential-expression results are provided in Supplementary Fig. S1b and Supplementary Table S2, respectively. Among maltose utilization genes, *malE*, *malP*, and *malQ* exhibited lower transcript abundance in the Δ*amyA* mutant relative to the wild type. Additional genes associated with maltose transport and metabolism, including *lamB*, *malF*, *malG*, *malK*, and *malS*, also showed negative log_2_ fold-change values (Fig. 7). These transcriptional changes were consistent with the delayed growth phenotype observed in maltose minimal medium (Fig. 4B), suggesting that loss of AmyA is associated with reduced expression of genes involved in maltose utilization. In contrast, several glycogen-related genes, including *glgC*, *glgA*, *glgP*, and *glgS*, displayed positive log_2_ fold-change values in the Δ*amyA* mutant, whereas *glgB* and *glgX* showed reduced transcript abundance relative to the wild type (Fig. 7). These transcriptional changes were accompanied by increased glycogen accumulation in the mutant (Figs. 4C and Fig. 5), indicating altered glycogen metabolism in the absence of AmyA. Taken together, these results suggest that loss of AmyA is associated with changes in maltose utilization and glycogen metabolism.

## Discussion

Cytoplasmic α-amylases have received considerably less attention than their extracellular counterparts, and their physiological functions remain poorly understood. The present study suggests that AmyA represents a distinct lineage of intracellular α-amylases that is structurally simpler than classical extracellular and maltogenic α-amylases. Unlike these enzymes, AmyA consists only of the conserved A-, B-, and C-domains and lacks additional domains associated with secretion, substrate binding, or specialized carbohydrate recognition [21]. Such a streamlined architecture is consistent with a role for AmyA in processing intracellular glucan intermediates rather than complex extracellular polysaccharides. The broad distribution of AmyA homologs among diverse bacterial taxa further suggests that intracellular GH13 enzymes may represent a conserved strategy for intracellular glucan metabolism across bacteria.

The biochemical properties of AmyA support a role in intracellular maltodextrin metabolism. AmyA hydrolyzed linear maltooligosaccharides and generated shorter glucans, including maltotriose. Interestingly, deletion of *amyA* produced a clear phenotype during growth on maltose but not on glucose. The distinct growth phenotypes observed with glucose and maltose may reflect differences in their uptake and intracellular metabolism. In *E. coli*, glucose is primarily taken up through the phosphotransferase system (PTS), whereas maltose is transported through the maltose/maltodextrin transport system and subsequently metabolized by intracellular maltodextrin-processing enzymes [5, 11]. This observation suggests that AmyA is not essential for growth on glucose, but rather contributes to the processing of intracellular maltodextrins generated during maltose utilization. Because maltooligosaccharides serve as key intermediates linking carbohydrate uptake and downstream metabolism, efficient turnover of these compounds may be important for maltose utilization. The delayed growth observed in the Δ*amyA* mutant is therefore consistent with a role for AmyA in facilitating adaptation to maltose as a carbon source. Intracellular maltodextrin metabolism in *E. coli* also involves MalQ and MalZ, which process maltodextrins through enzymatic activities distinct from that of AmyA [11]. The presence of these enzymes may partially compensate for the loss of AmyA, although the functional relationship between AmyA and these maltodextrin-metabolizing enzymes remains to be determined.

One of the most notable findings of this study was the substantial increase in glycogen accumulation in the Δ*amyA* mutant. This phenotype highlights the close metabolic connection between intracellular maltodextrin metabolism and glycogen homeostasis. Previous studies have shown that disruption of MalP also results in excessive glycogen accumulation, indicating that efficient turnover of intracellular glucans is important for regulating carbon storage [14]. Although AmyA catalyzes a distinct reaction, the similar glycogen-overaccumulation phenotype observed in the Δ*amyA* mutant further supports a functional link between maltodextrin processing and glycogen metabolism. However, because glycogen accumulation reflects the balance between synthesis and degradation, the present results do not establish which process is primarily responsible for the increased glycogen level. In addition to increased glycogen accumulation, the Δ*amyA* mutant exhibited a shift in glycogen chain-length distribution, characterized by an increased proportion of intermediate-length chains and a reduced proportion of longer chains. These results suggest that loss of AmyA is associated not only with increased glycogen accumulation but also with changes in glycogen architecture. Recent studies have similarly demonstrated that perturbation of glycogen-metabolizing enzymes can alter glycogen molecular structure in *E. coli* [22]. Interestingly, similar changes in glycogen chain-length distribution were observed in the Δ*amyA* mutant under both glucose and maltose conditions, despite the distinct effects of *amyA* deletion on growth. Together, these observations support a role for AmyA in maintaining intracellular glucan homeostasis.

The transcriptomic and physiological data provide a possible explanation for these phenotypes. Several genes associated with maltose utilization showed reduced transcript abundance in the Δ*amyA* mutant, whereas multiple glycogen-related genes exhibited altered expression patterns. AmyA generates short maltooligosaccharides, including maltose and maltotriose, during maltodextrin hydrolysis, and maltotriose is known to function as a physiological inducer of MalT, the master regulator of the maltose regulon [12, 13]. Thus, AmyA-mediated production of short maltooligosaccharides may influence MalT-dependent regulation of maltose-utilization genes. This interpretation is consistent with both the delayed growth phenotype observed in maltose minimal medium and the reduced transcript abundance of *malE*, *malP*, *malQ*, and other maltose-utilization genes. Increased glycogen accumulation in the Δ*amyA* mutant further indicates altered glycogen metabolism associated with the loss of AmyA. Based on these observations, we propose a model in which AmyA may link intracellular maltodextrin processing and glycogen metabolism through the generation of short maltooligosaccharides that facilitate efficient maltose utilization (Fig. 8). Taken together, these findings support a physiological role for AmyA in intracellular maltodextrin metabolism and suggest a relationship between AmyA function, maltose utilization, and glycogen metabolism in *E. coli*. These findings provide a useful framework for further investigation of the physiological functions of cytoplasmic α-amylases and their roles in intracellular carbohydrate metabolism.

## Supplemental Materials

Supplementary data for this paper are available on-line only at http://jmb.or.kr.



## Figures and Tables

**Fig. 1:**
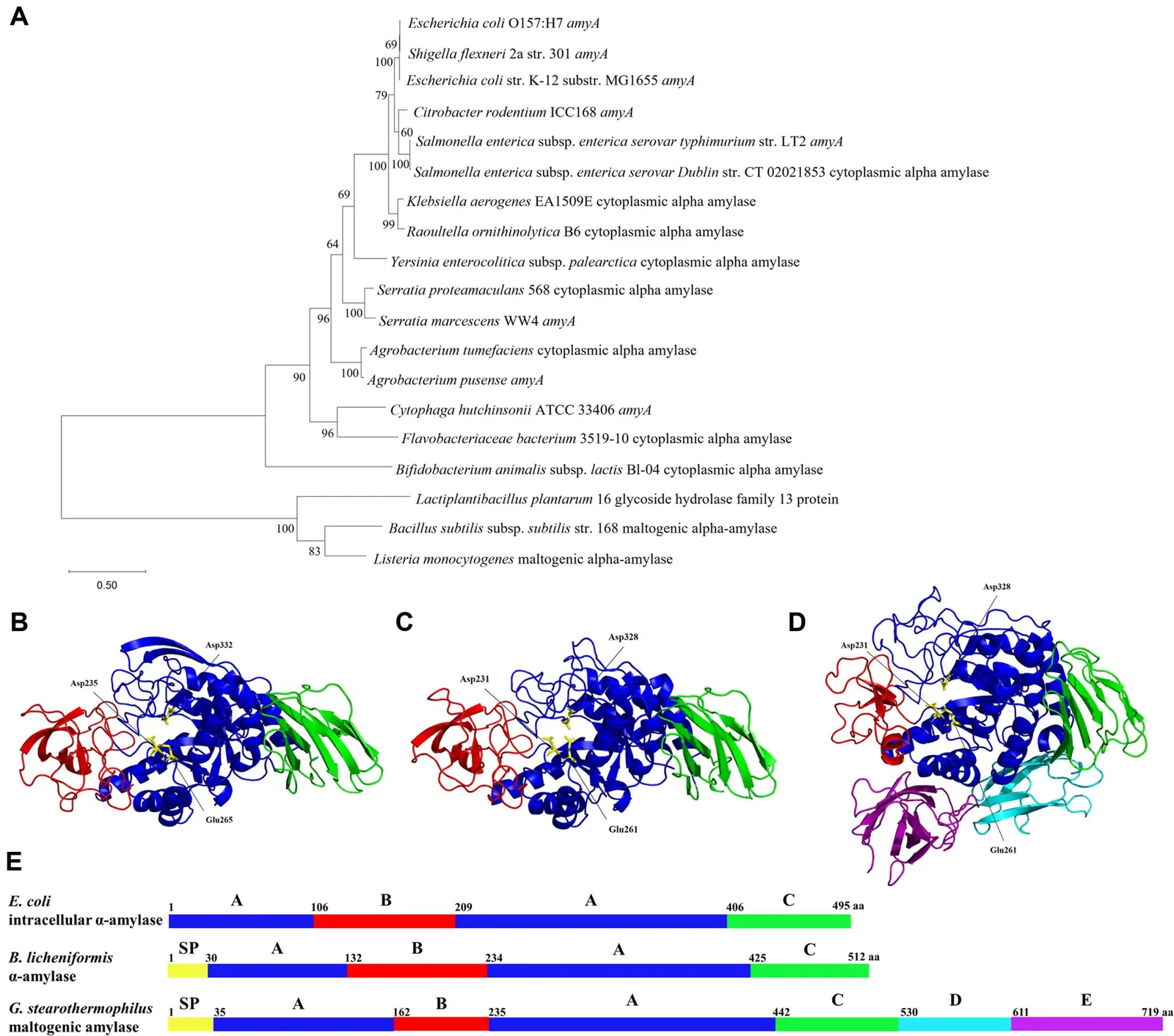
Phylogenetic analysis of AmyA homologs and related GH13 α-amylases. Amino acid sequences were retrieved from the NCBI database, and the phylogenetic tree was constructed using the Maximum Likelihood method with the WAG+G+I substitution model in MEGA 12. The reliability of the inferred tree topology was assessed by 1,000 bootstrap replicates, and bootstrap values (%) are shown at the nodes (**A**). AmyA homologs were identified in phylogenetically diverse bacterial taxa, including members of the Proteobacteria, Actinobacteria, and Bacteroidota, forming a cluster distinct from the maltogenic α-amylases included in the analysis. Structural comparison of *E. coli* AmyA obtained from the AlphaFold Protein Structure Database (AF-P26612-F1) (**B**), *B. licheniformis* α-amylase (PDB: 1BLI; **C**), and *B. stearothermophilus* maltogenic amylase (PDB: 1QHP; **D**). Structures are shown in the same orientation and colored according to domain organization (A-domain, blue; B-domain, red; C-domain, green). Additional D- and E-domains (cyan and purple, respectively) are present only in the maltogenic amylase. The conserved catalytic Asp–Glu–Asp residues are indicated as yellow sticks in each structure. Linear domain architectures are drawn to scale (**E**), highlighting that AmyA consists solely of the catalytic A/B/C domains and lacks both an N-terminal signal peptide and the accessory D- and E-domains present in the maltogenic α-amylase.

**Fig. 2:**
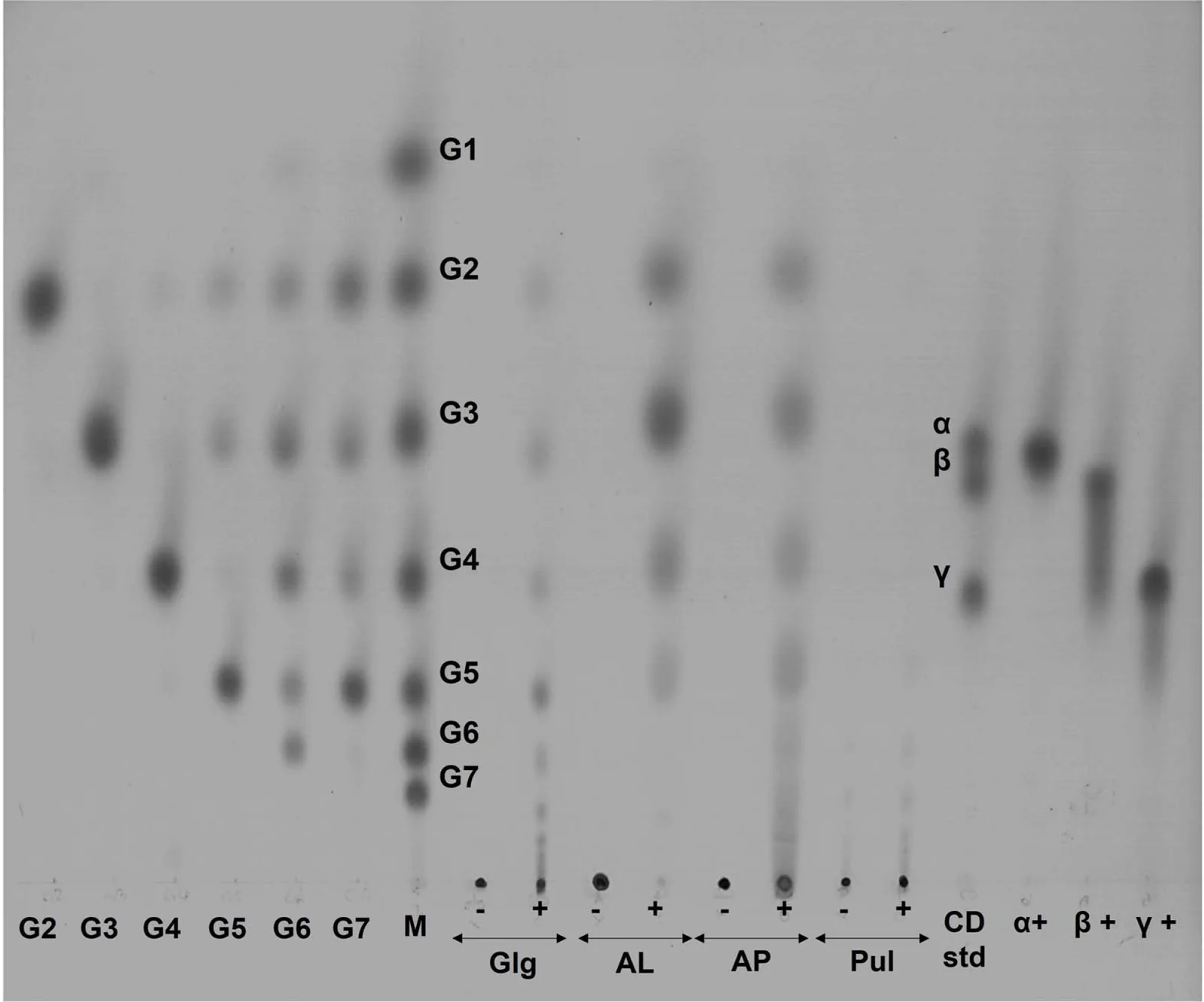
Substrate specificity of the cytoplasmic α-amylase AmyA analyzed by thin-layer chromatography (TLC). Linear maltooligosaccharides (G2–G7), glycogen (Glg), amylose (AL), amylopectin (AP), pullulan (Pul), and cyclodextrins (α-, β-, and γ-CD) were incubated with purified AmyA for 16 h. Lane M contains maltooligosaccharide standards (G1–G7). Reactions performed in the absence (−) or presence (+) of AmyA are indicated.

**Fig. 3:**
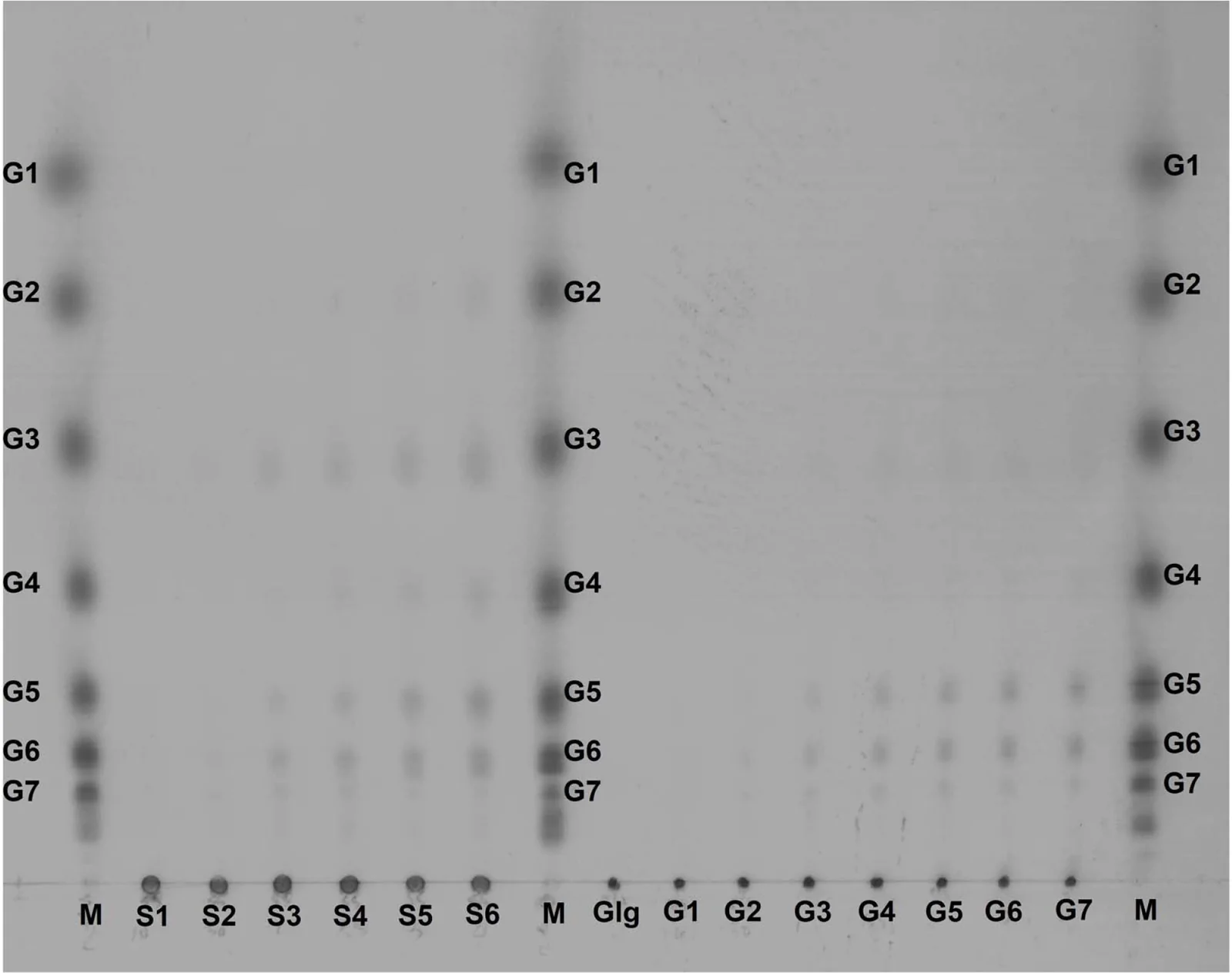
Time-course hydrolysis of soluble starch and glycogen by AmyA analyzed by thin-layer chromatography (TLC). Soluble starch was sampled after 10 min (S1), 30 min (S2), 1 h (S3), 1.5 h (S4), 3 h (S5), and 4 h (S6) of incubation. Glycogen was analyzed before incubation (Glg) and after 10 min (G1), 30 min (G2), 1 h (G3), 1.5 h (G4), 2 h (G5), 3 h (G6), and 4 h (G7) of incubation. Lane M contains maltooligosaccharide standards (G1–G7).

**Fig. 4:**
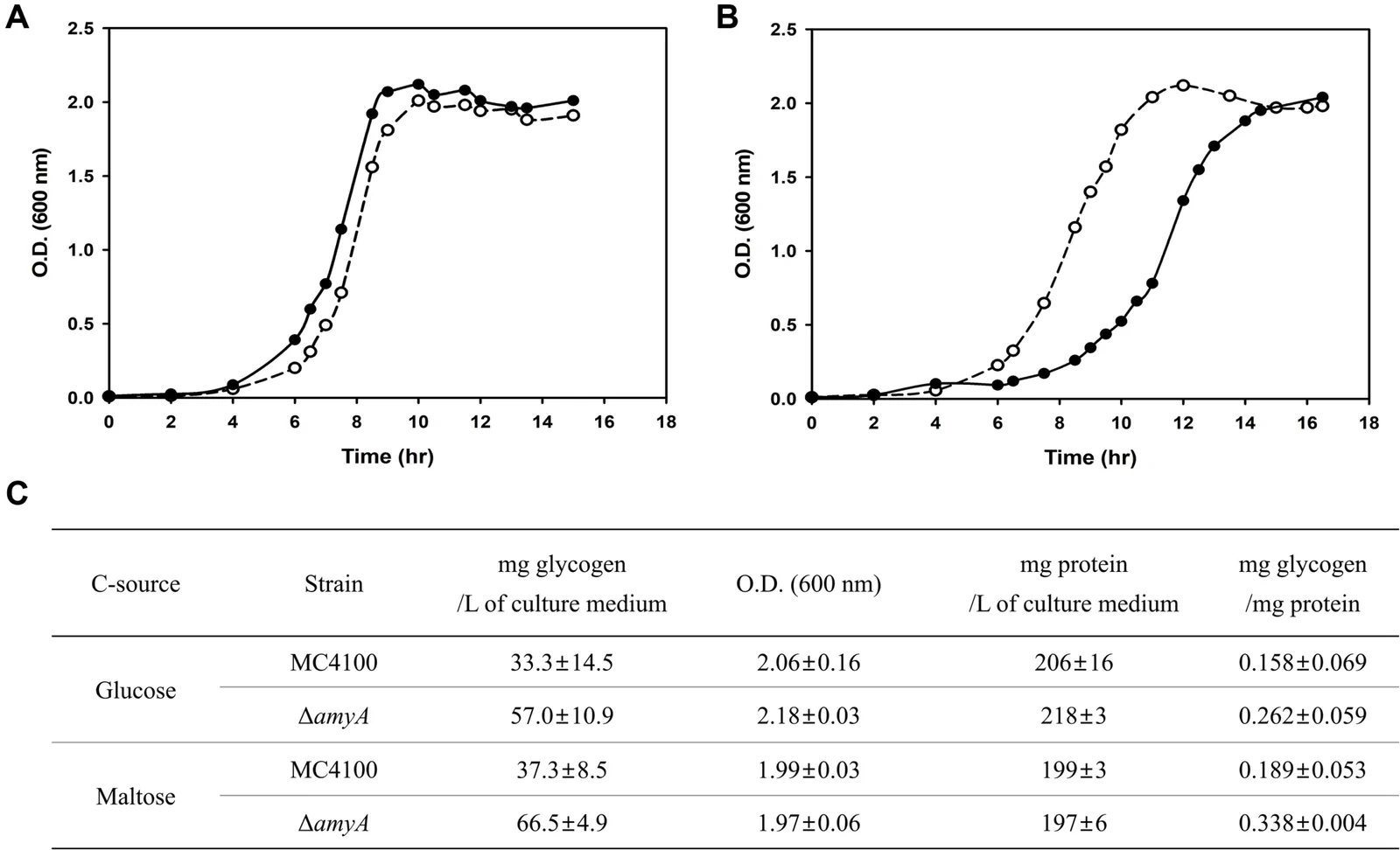
Effects of *amyA* deletion on growth and glycogen accumulation in *E. coli*. (**A**) Representative growth curves of the Δ*amyA* mutant (●) and wild-type strain ( ○ ) cultured in glucose minimal medium. (**B**) Representative growth curves of the Δ*amyA* mutant ( ● ) and wild-type strain ( ○ ) cultured in maltose minimal medium. Growth assays were performed independently three times with similar results, and representative growth curves are presented. (**C**) Intracellular glycogen levels measured at the early stationary phase in cells grown in glucose and maltose minimal media. Values are presented as mean ± SD from three independent experiments (n = 3). Cell growth was monitored by measuring optical density at 600 nm (OD_600_).

**Fig. 5:**
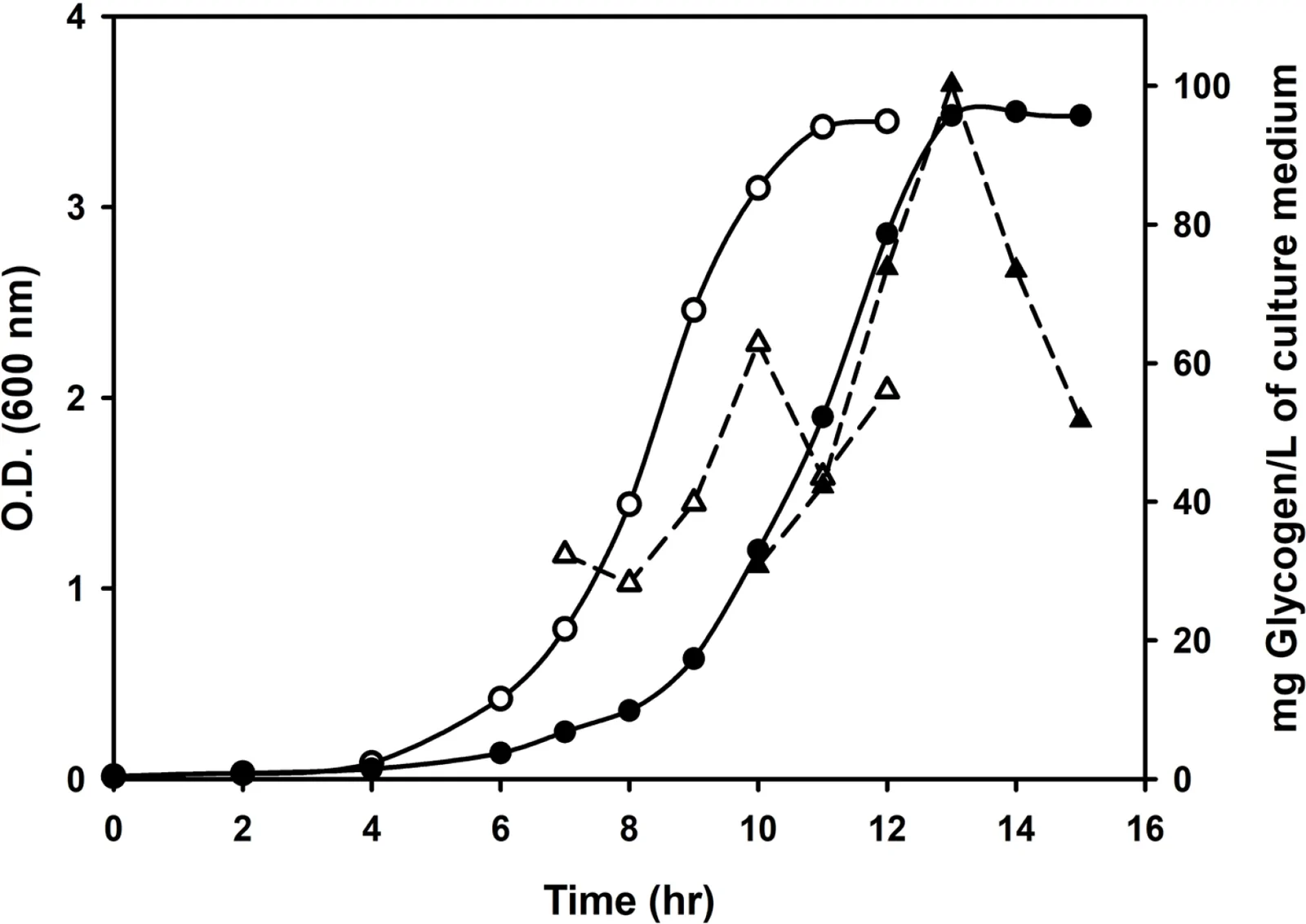
Time-course analysis of cell growth and glycogen accumulation in wild-type and Δ*amyA* strains cultured in G2 medium. Cell growth (circles) and intracellular glycogen levels (triangles) were monitored throughout cultivation in G2 medium. Open symbols indicate the wild-type strain, and closed symbols indicate the Δ*amyA* mutant. Glycogen levels are expressed as mg/L of culture medium. Experiments were performed independently three times, and a representative experiment is shown.

**Fig. 6:**
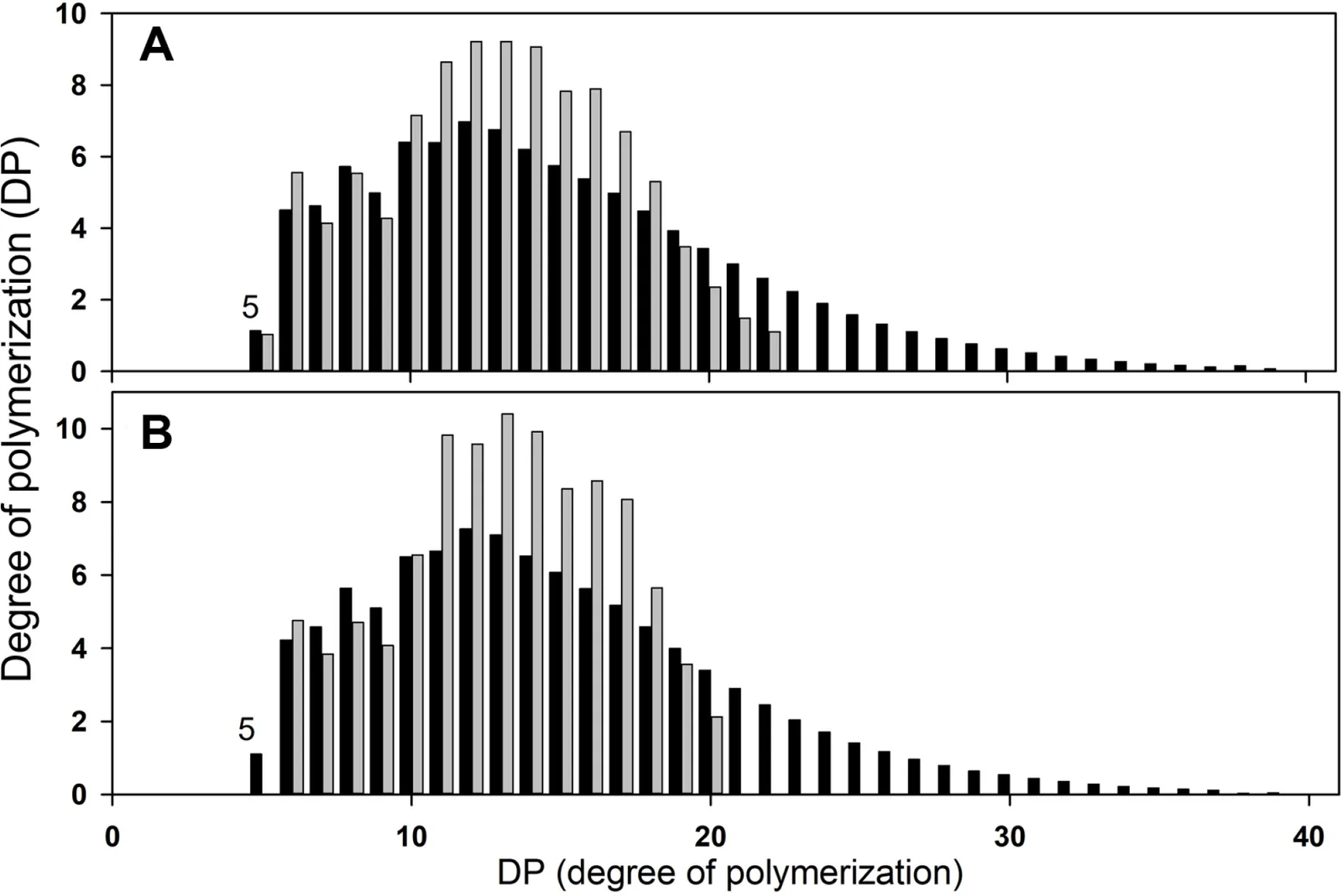
Chain-length distribution of glycogen isolated from MC4100 (black bars) and the Δ*amyA* mutant (gray bars). Normalized degree-of-polymerization (DP) profiles of debranched glycogen are shown for cells grown in (**A**) glucose minimal medium and (**B**) maltose minimal medium. Experiments were performed independently three times with similar results, and representative profiles are shown.

**Fig. 7:**
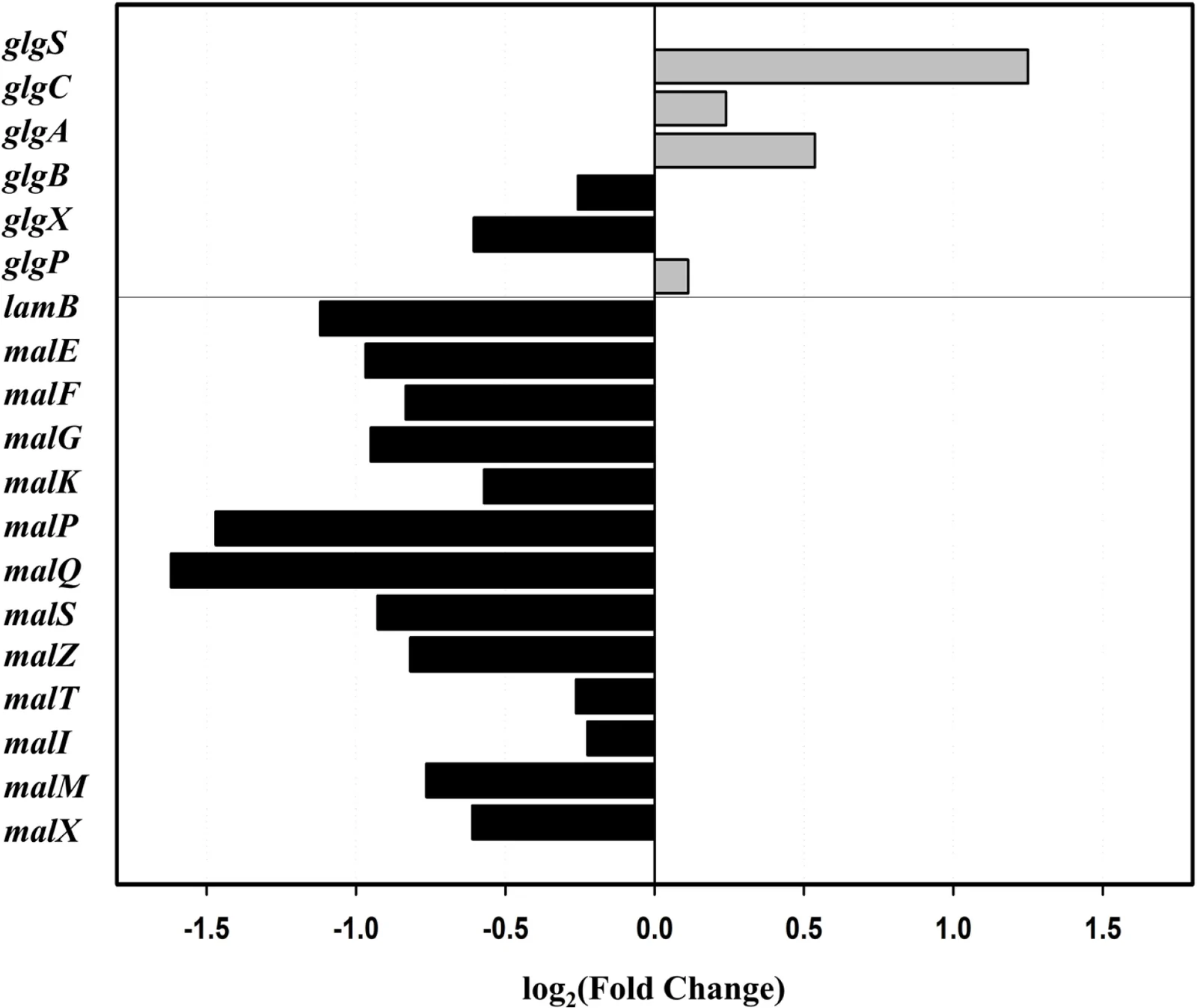
Expression profiles of glycogen metabolism (*glg*) and maltose utilization (*mal*) genes in the Δ*amyA* mutant relative to the wild-type strain. Bars represent log_2_ fold changes calculated from mean FPKM values of two biological replicates. Gray and black bars indicate positive and negative log_2_ fold changes, respectively. Genes are grouped according to their functional categories. Replicate FPKM values and differential-expression statistics are provided in Supplementary Table S1.

**Fig. 8:**
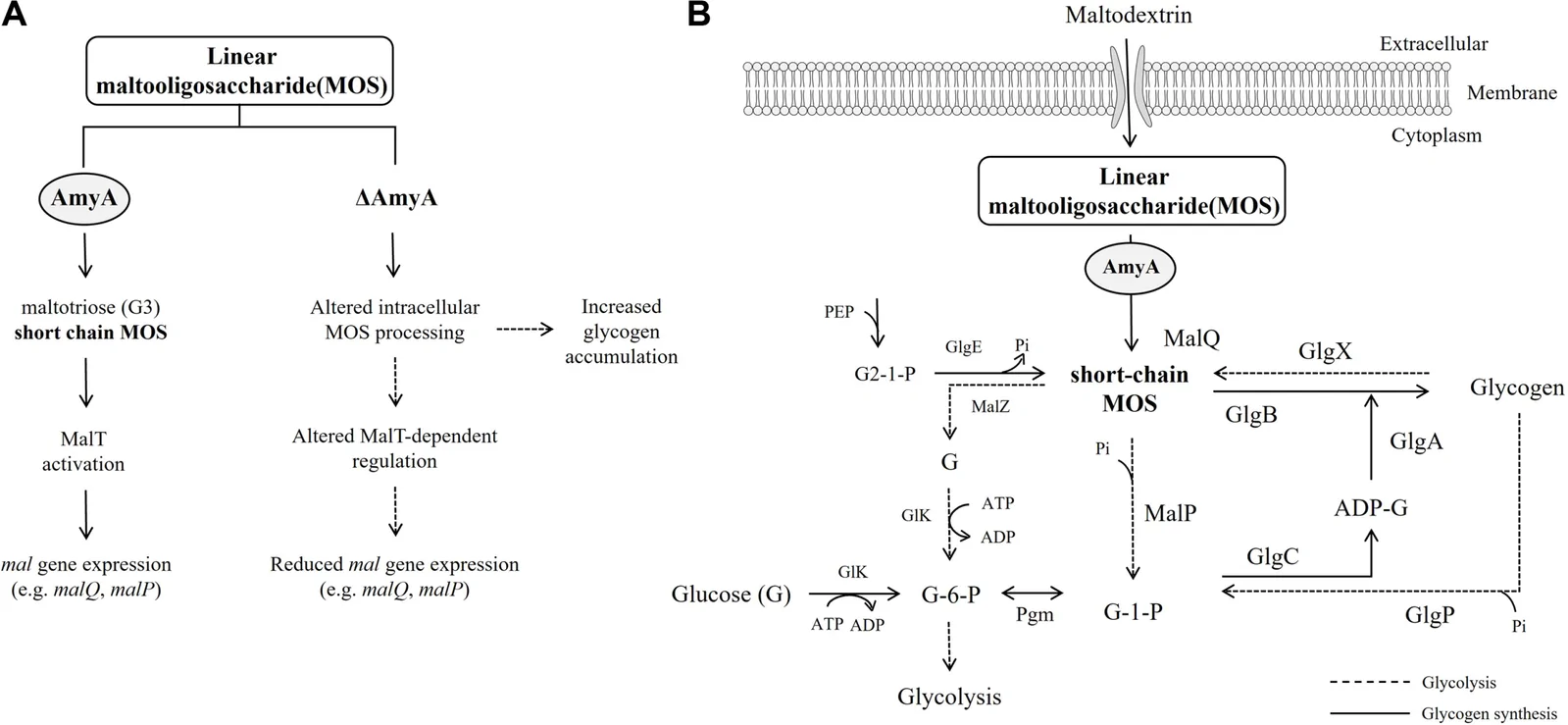
Proposed model for the physiological role of AmyA in intracellular maltodextrin and glycogen metabolism. (**A**) Proposed effects of AmyA activity and *amyA* deletion on intracellular maltooligosaccharide processing, MalT-dependent regulation, and glycogen accumulation. AmyA generates short-chain maltooligosaccharides, including maltotriose (G3), which may contribute to MalT-dependent regulation of maltose-utilization genes. In the Δ*amyA* mutant, altered intracellular maltooligosaccharide processing is proposed to be associated with reduced expression of *mal* genes and increased glycogen accumulation. Dashed arrows in panel (**A**) indicate proposed relationships. (**B**) Proposed pathway linking intracellular maltooligosaccharide metabolism and glycogen turnover. AmyA is proposed to generate short-chain maltooligosaccharides that may enter glycolytic pathways through MalQ- and MalP-mediated reactions or participate in glycogen metabolism. In panel (**B**), solid and dashed arrows indicate glycogen synthesis and glycolytic pathways, respectively.
