# Supplementary material for: Cytoplasmic α-Amylase AmyA Modulates Maltodextrin/Glycogen Metabolism in Escherichia coli
Source: J Microbiol Biotechnol. 2026 Sep 4;36:e2606044. doi: 10.4014/jmb.2606.06044 (PMC13754560; doi:10.4014/jmb.2606.06044)
Supplement: Supplementary file 1 [file jmb-36-e2606044-supple.pdf]

J. Microbiol. Biotechnol. 2026. 36: e2606044
https://doi.org/10.4014/jmb.2606.06044

(a)

Sample	Total reads	Genome-mapped reads	Mapping rate (%)	Properly paired reads	Properly paired rate (%)
**WT-1**	23,349,594	16,181,997	69.30	2,736,164	11.72
**WT-2**	19,601,190	12,405,795	63.29	1,868,022	9.53
**Δ*amyA*-1**	21,432,488	15,796,635	73.70	2,675,866	12.49
**Δ*amyA*-2**	24,491,264	17,617,889	71.94	2,991,918	12.22

(b)

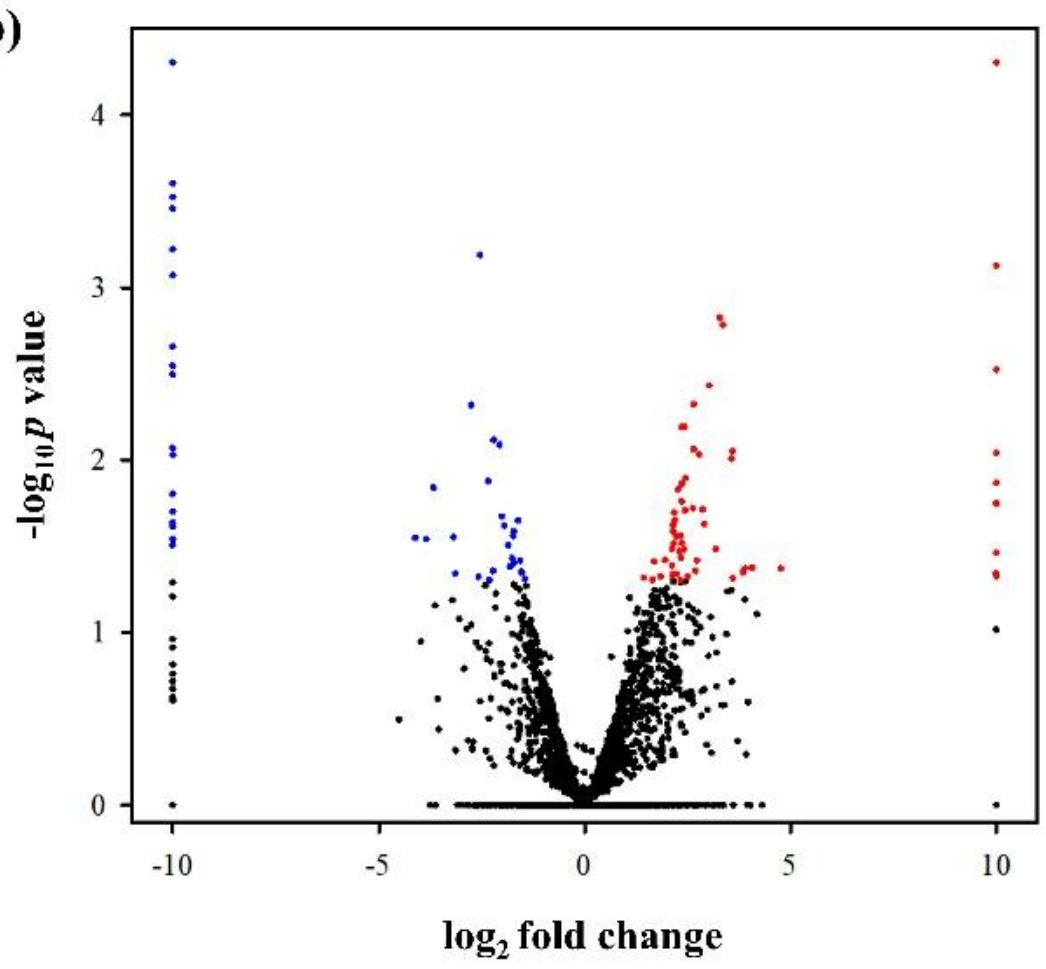

Fig. S1. RNA-seq sequencing statistics and genome-wide differential gene expression analysis of wild-type and Δ*amyA* strains. (a) Summary of sequencing and genome-mapping statistics for two biological replicates of the WT and Δ*amyA* strains. (b) Volcano plot showing genome-wide gene expression changes in the Δ*amyA* strain relative to the WT strain. The x-axis represents the $\log_2$ fold change (Δ*amyA*/WT), and the y-axis represents $-\log_{10}$(*p* value). Genes with a nominal $p < 0.05$ are shown in red for up-regulated genes and blue for down-regulated genes, whereas genes with $p \geq 0.05$ are shown in black. Infinite $\log_2$ fold-change values were plotted at the corresponding axis limits (+10 or −10) for visualization. Multiple-testing-adjusted *q* values (FDR) for all genes are provided in Supplementary Table S2.

Table S1. Transcript abundance and statistical parameters for genes presented in Figure 7.

Category	Gene	Gene ID	WT-1 FPKM	WT-2 FPKM	WT mean FPKM	Δ*amyA*-1 FPKM	Δ*amyA*-2 FPKM	Δ*amyA* mean FPKM	$\log_2$ fold change	*p* value	FDR (*q* value)
Glycogen metabolism	*glgS*	TBIG002958	84.370	131.856	108.113	115.198	381.760	248.479	1.25	0.16790	0.987370
Glycogen metabolism	*glgC*	TBIG003327	13.936	38.620	26.278	13.320	46.424	29.872	0.239	0.84255	0.987370
Glycogen metabolism	*glgA*	TBIG003326	13.838	23.449	18.643	14.098	38.005	26.052	0.537	0.68125	0.987370
Glycogen metabolism	*glgB*	TBIG003329	15.680	43.800	29.740	11.490	37.183	24.336	-0.257	0.80190	0.987370
Glycogen metabolism	*glgX*	TBIG003328	4.491	28.523	16.507	7.259	14.379	10.819	-0.606	0.75945	0.987370
Glycogen metabolism	*glgP*	TBIG003325	14.172	21.418	17.795	9.340	26.739	18.039	0.112	0.88950	0.987370
Maltose utilization	*lamB*	TBIG003939	314.786	349.934	332.360	158.222	152.363	155.293	-1.12	0.14805	0.987370
Maltose utilization	*malE*	TBIG003937	643.878	638.359	641.119	395.438	281.803	338.621	-0.968	0.13310	0.987370
Maltose utilization	*malF*	TBIG003936	56.619	86.818	71.719	21.568	58.260	39.914	-0.834	0.28765	0.987370
Maltose utilization	*malG*	TBIG003935	33.542	58.126	45.834	14.615	31.199	22.907	-0.951	0.26590	0.987370
Maltose utilization	*malK*	TBIG003938	189.294	213.903	201.599	193.337	87.872	140.604	-0.572	0.48375	0.987370
Maltose utilization	*malP*	TBIG003316	220.249	272.691	246.470	113.072	70.966	92.019	-1.47	0.06230	0.979808
Maltose utilization	*malQ*	TBIG003315	103.347	132.175	117.761	33.029	40.760	36.894	-1.62	0.05555	0.979808
Maltose utilization	*malS*	TBIG003474	23.972	41.672	32.822	20.749	13.796	17.273	-0.928	0.21260	0.987370
Maltose utilization	*malZ*	TBIG000385	21.812	32.856	27.334	19.867	10.900	15.383	-0.819	0.28335	0.987370
Maltose utilization	*malT*	TBIG003317	20.759	21.088	20.924	19.280	15.160	17.220	-0.263	0.69930	0.987370
Maltose utilization	*malI*	TBIG001557	2.899	5.142	4.021	5.049	1.844	3.446	-0.225	0.80610	0.987370

Maltose utilization	*malM*	TBIG003940	34.475	62.073	48.274	13.560	42.620	28.090	-0.765	0.34220	0.987370
Maltose utilization	*malX*	TBIG001558	5.484	0.856	3.170	2.789	1.318	2.053	-0.611	1.00000	1.000000

Note: Values are derived from two biological replicates for each strain (WT, $n = 2$; Δ*amyA*, $n = 2$). FPKM, fragments per kilobase of transcript per million mapped reads. $\log_2$ fold-change, p values, and FDR-adjusted q values were obtained from the differential-expression analysis. Replicate-specific FPKM values are shown for reference.
